# Phantom Validation of Tc-99m Absolute Quantification in a SPECT/CT Commercial Device

**DOI:** 10.1155/2016/4360371

**Published:** 2016-12-14

**Authors:** Silvano Gnesin, Paulo Leite Ferreira, Jerome Malterre, Priscille Laub, John O. Prior, Francis R. Verdun

**Affiliations:** ^1^Institute of Radiation Physics, Lausanne University Hospital, Lausanne, Switzerland; ^2^Department of Nuclear Medicine and Molecular Imaging, Lausanne University Hospital, Lausanne, Switzerland

## Abstract

*Aim*. Similar to PET, absolute quantitative imaging is becoming available in commercial SPECT/CT devices. This study's goal was to assess quantitative accuracy of activity recovery as a function of image reconstruction parameters and count statistics in a variety of phantoms.* Materials and Methods*. We performed quantitative ^99m^Tc-SPECT/CT acquisitions (Siemens Symbia Intevo, Erlangen, Germany) of a uniform cylindrical, NEMA/IEC, and an anthropomorphic abdominal phantom. Background activity concentrations tested ranged: 2–80 kBq/mL. SPECT acquisitions used 120 projections (20 s/projection). Reconstructions were performed with the proprietary iterative conjugate gradient algorithm. NEMA phantom reconstructions were obtained as a function of the iteration number (range: 4–48). Recovery coefficients, hot contrast, relative lung error (NEMA phantom), and image noise were assessed.* Results*. In all cases, absolute activity and activity concentration were measured within 10% of the expected value. Recovery coefficients and hot contrast in hot inserts did not vary appreciably with count statistics. RC converged at 16 iterations for insert size > 22 mm. Relative lung errors were comparable to PET levels indicating the efficient integration of attenuation and scatter corrections with adequate detector modeling.* Conclusions*. The tested device provided accurate activity recovery within 10% of correct values; these performances are comparable to current generation PET/CT systems.

## 1. Introduction

Positron emission tomography (PET) is currently considered the gold-standard modality for absolute quantification in emission tomography. In contrast, up to now, single photon emission computed tomography (SPECT) has been used mostly for qualitative and semiquantitative clinical investigations; by consequence the signal at the pixel level (in units of counts) is proportional to the number of measured events.

Compared to PET, SPECT suffers from inferior spatial resolution and sensitivity but enables metabolic studies with long half-life gamma emitter radioisotopes that are well-suited to match the underlying tracer metabolism and biodistribution of target processes over hours or days, in addition to its capability of simultaneous multi-isotope imaging [1].

Similar to PET, absolute quantitative imaging has recently become available in commercial SPECT/CT devices [2]. A list of potential uses of quantitative SPECT in a clinical setting has been described extensively in the publication by Bailey and Willowson [3].

One of the most important fields of application of quantitative SPECT imaging is internal dosimetry. In the recent past, internal dosimetry has been successfully accomplished with SPECT-only cameras [4]; however, the latest generation of hybrid SPECT/CT scanners offer significant computational advantages thanks to the implementation of attenuation, scatter, resolution recovery, and dead time corrections [5, 6]. The implementation of cross calibration procedures for specific radioisotope/collimator combinations can achieve absolute quantification in terms of Bq/cm^3^. Quantitative SPECT calibration can be achieved by performing appropriate phantom studies as reported by Zeintl et al. [7]. Such procedures should be repeated before each quantitative patient scan and it is not supported by the commercial device.

Such quantitative performances, in assessing ^99m^Tc activity concentration, in a ready-to-use environment are claimed by the newly developed Siemens Symbia Intevo SPEC/CT. In particular the manufacturer claims a <10% accuracy deviation in assessing ^99m^Tc activity concentration using the proprietary, low-energy, high-resolution collimator.

The goal of this study was to assess the quantitative accuracy of activity recovery as a function of image reconstruction parameters and count statistics, first in a basic cylindrical phantom and then in more anthropomorphic geometries which mimic clinically relevant setups.

## 2. Material and Methods

Quantitative ^99m^Tc-SPECT/CT acquisitions of a cylindrical homogeneous phantom, a NEMA/IEC phantom, and an anthropomorphic abdominal phantom were performed on a Siemens Symbia Intevo SPECT/CT device (Siemens, Erlangen, Germany) equipped with a low-energy, high-resolution collimator. SPECT acquisitions were acquired with 120 projections (20 s/projection) and reconstructed using the proprietary iterative conjugate gradient algorithm on a Siemens Syngo work station. CT-based attenuation correction and dual-energy window scatter correction were systematically applied in SPECT reconstructions. The photopeak emission energy window for ^99m^Tc was set to 129–150 keV, while the lower scatter window was set to 108–129 keV. SPECT emission data were decay-corrected to the time of activity administration; for each phantom study this corresponded to the time of the calibration of the activity used for the phantom preparation. Quantitative accuracy of the system was verified with a monthly periodicity using a ^57^Co point source (whose nominal activity was certified by the National Institute of Standard and Technology or NIST, Gaithersburg, MD) for the system energy and sensitivity calibration according to the manufacturer's specifications.

### 2.1. Different Phantom Studies Were Performed

#### 2.1.1. Cylindrical Homogeneous Phantom

We started our investigation by assessing quantitative accuracy in terms of activity concentration recovery in a simple geometric configuration consisting of a uniform background (cylindrical homogenous phantom). The cylindrical phantom was 18 cm long and 20 cm in diameter for an inner volume of 5683 mL. The SPECT reconstruction for the cylindrical phantom employed 24 iterations, 4 subsets, and a Gaussian smoothing with a full width at half maximum (FWHM) of 7.5 mm. This represents the standard quantitative reconstruction algorithm in our clinical setup. The accuracy was evaluated by a calibration factor (Bg. cal) which was the ratio between the activity concentration measured in the reconstructed SPECT phantom background (*a*_*c*,bg_) and the expected activity concentration (*A*_*c*,bg_) known from the phantom preparation:

(1) $$\begin{array}{c} Bg.cal=\frac{a_{c,bg}}{A_{c,bg}}. \end{array}$$

**Figure 1:**
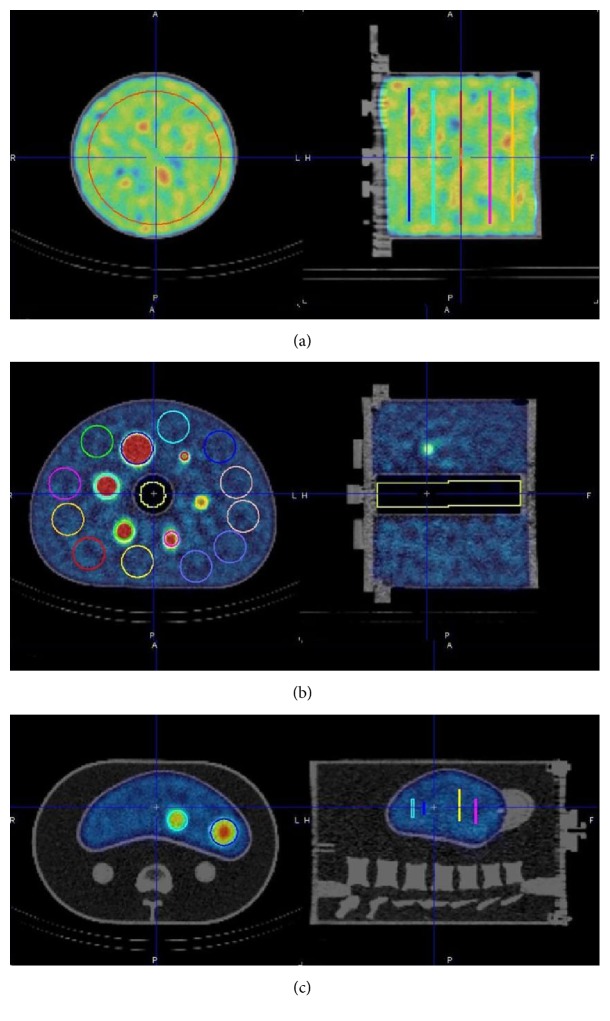
Cross section of the tree phantom employed for this study: (a) cylindrical phantom, (b) NEMA/IEC NU2 phantom, and (c) Kyoto liver anthropomorphic phantom.

*a*_*c*,bg_ was evaluated as the average on 5 circular region of interests of 16 cm diameter centered on the cylinder axis placed at different axial locations (Figure 1(a)). The image noise was evaluated by the coefficient of variation (COV), which is the ratio between the standard deviation and the average signal measured from the phantom background:

(2) $$\begin{array}{c} COV\left(\;\%\;\right)=\frac{\sigma_{bg}}{a_{c,bg}}\times 100. \end{array}$$

To test the influence of the count statistics on quantitative accuracy, we performed successive SPECT acquisitions at different time points. The total activity in the phantom and thus the background activity concentration decreased as a function of time elapsed between the phantom preparation and the actual acquisition time. The nominal activity concentration in the phantom background at the time of the acquisition, the calibration factor, and the coefficient of variation are reported in Table 1.

#### 2.1.2. NEMA/IEC Phantom

A NEMA/IEC NU2 phantom was also used to test the quantitative accuracy in a somehow more anthropomorphic configuration; in particular this phantom makes it possible to assess recovery coefficients as well as hot contrast in spherical inserts as a function of the insert size.

Six spherical inserts 10, 13, 17, 22, 28, and 37 mm in diameter were filled with an activity concentration (*A*_*c*,sph_) 8.5 times higher than the activity concentration present in the phantom background (as reported in Table 2). A lung insert was also available for this phantom and used to estimate a relative lung error (Δ*C*_lung_) (see Figure 1(b)).

For each spherical insert (*j*) the recovery coefficients (RC) were defined by

(3) RCj,max=acsph,j,maxAc,sph,RCj,A50=acsph,j,A50Ac,sph,

where *a*_*c*,sph,*j*,max_ is the measured maximum voxel value (in terms of activity concentration) for a given spherical insert (*j* = 1 to 6) and *a*_*c*,sph,*j*,A50_ is the average voxel value for each hot insert volume of interest (VOI) defined by a 3D isocontour at 50% adapted for background as defined in [8] and recommended by the EANM Guidelines for FDG tumor PET imaging [9].

According to standard National Electrical Manufacturers Association (NEMA) NU2 standard, the hot contrast (*Q*_*H*_) was defined by

(4) $$\begin{array}{c} Q_{H,j}\left(\;\%\;\right)=\frac{\left(a_{c,sph,j}/a_{c,bg}\right)-1}{\left(A_{c,sph}/A_{c,bg}\right)-1}\times 100, \end{array}$$

while inserting the relative error in the lung insert was defined by:

(5) $$\begin{array}{c} \Delta C_{\text{lung}}\left(\;\%\;\right)=\frac{a_{c,\text{lung}}}{A_{c,bg}}\times 100, \end{array}$$

where *a*_*c*,lung_ is the average activity concentration measured in a cylindrical volume of interest 3 cm in diameter and 16 cm in length placed into the lung insert.

SPECT reconstructions of NEMA/IEC NU2 phantom acquisition Scan 1 were performed by varying the iteration number (iteration range: 4–48, 4 subsets, Gaussian smoothing of FWHM 7.5 mm) to study the impact of this parameter on recovery coefficients (RC), hot contrast relative lung error, and image noise (COV).

Background activity concentration (AC_bg_) as well as activity concentration in spherical inserts were decreasing as a function of time between successive SPECT acquisitions of the NEMA phantom as summarized in Table 2, where 16 iterations were used for the reconstruction.

#### 2.1.3. Kyoto Liver Anthropomorphic Phantom

Lastly, we assessed quantitative accuracy using an anthropomorphic abdominal phantom (commercial Kyoto Liver/Kidney phantom, Nuclemed, Roeselare, Belgium) that includes a liver insert (volume = 1.8 L) with 3 hot-spheres (20–30–40 mm diameter, 5.2 : 1 hot-sphere to liver activity concentration ratio) and a high-density element such as a lumbar spine insert (Figure 1(c)). We assessed the hot contrast for the three spherical inserts.

Liver phantom reconstructions were performed using 16 iterations and 4 subsets (FWHM = 7.5 mm); the only variable was the time elapsed between the phantom preparation and the SPECT acquisition to evaluate quantitative accuracy as a function of counting statistics.

For the three phantom configurations tested, the total activity in the reconstructed field of view (that contains the whole phantom, total background plus overall insert activity) was also evaluated and compared to the nominal total activity known from the phantom preparation as reported in Table 3.

Image coregistrations of SPECT and CT data as well as volume of interest segmentation (based on CT data) were performed using PMOD (release 3.4) software (PMOD Technologies Ltd., Zurich, Switzerland).

In all phantom acquisitions the total recovered activity (*A*_tot,rec_) in the field of view was compared to the total activity (*A*_tot_) known at the phantom preparation to derive the total activity deviation:

(6) $$\begin{array}{c} \Delta A_{tot}\left(\;\%\;\right)=\frac{A_{tot,rec}-A_{tot}}{A_{tot}}\times 100. \end{array}$$

## 3. Results and Discussion

As reported in Tables 1, 2, and 3, the background activity concentration for the different phantom configurations we tested exhibited overall good quantitative accuracy. The measured values were within 10% of the expected values, even at low activity concentrations (<10 kBq/mL), thus in agreement with the accuracy level (<10%) stated by the manufacturer. As expected, the COV increased with decreasing statistics.

**Figure 2:**
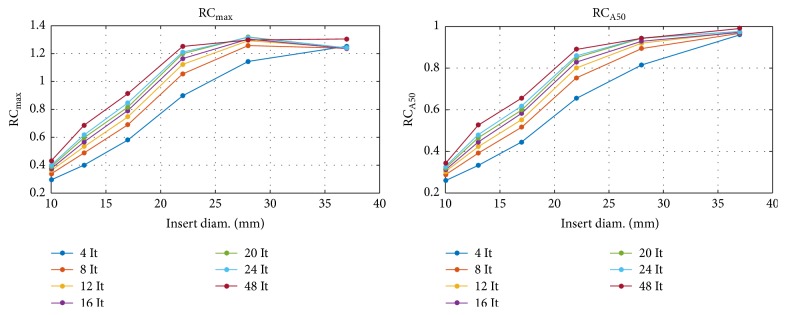
Recovery coefficient (RC_max_ and RC_A50_) as a function of the insert diameter with a different number of iterations (It range: 4–48) for the iterative reconstruction algorithm using the NEMA/IEC phantom.

For the NEMA phantom (NEMA Scan 1), RC values were comparable to PET derived reference levels reported in the EANM guidelines for FDG PET for tumor imaging: version 1.0 [9]. Nevertheless, because of the inferior intrinsic spatial resolution of SPECT compared to PET, RC values in small volumes remain lower than present generation time-of-flight (TOF) PET adopting resolution recovery methods such as the point spread function correction. As shown in Figure 2, RC values increased less than linearly as a function of the iteration number. The RC values for a 22 mm hot insert showed a relative increment (mean/max) of 13/18%, 8.5/12%, and 4/7.5% when varying from It = 4 to 8, 12 to 24, and 24 to 48, respectively.

The convergence for activity concentration recovery in insert with diameter > 24 mm was satisfactory (less than 5% variation) for It ≥ 16, while more iterations are suitable for activity recoveries in smaller volumes.

The lung relative error (Table 2) was less than 10% for tested background activity concentrations > 12 kBq/mL. Such lung error values are comparable to what can be obtained in ^18^F-FDG TOF PET phantom scans [10]. The lung relative error increased with lower statistic and was found to be approximately 20% for *A*_*c*,bg_ of 1.5 kBq/mL (NEMA Scan 4).

Recovery coefficients and hot contrast (*Q*_*H*_) measured in NEMA (Table 2) and liver phantom (Table 3) scans with a fixed reconstruction setting (It = 16, Ss = 4, and FWHM = 7.5 mm) did not vary appreciably with count statistics. The total activity contained in the SPECT field of view was measured for each phantom scan and the quantitative agreement with the actual activity contained in the phantom was within 9% (range [+3.5% to 8.6%]) for the cylindrical uniform phantom, 5% (range [−0.1% to +4%]) for the NEMA phantom, and 6% (range [−1% to +5.8%]) for the liver phantom of the true total activity.

**Figure 3:**
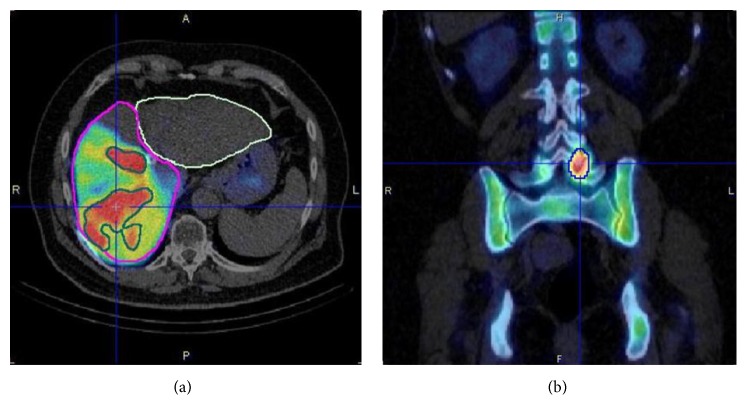
(a) ^99m^Tc-MAA SPECT/CT prior to Y-90 radioembolisation after administration of 105.8 MBq of ^99m^Tc-MAA to the right lobe of the liver (volume = 1290 mL, segmentation in violet). In this specific case the recovered total activity on the SPECT field of view was 98.2 MBq that is 7% less than the total administered activity. Average activity concentration in the normal parenchyma was 35.6 kBq/ml (range: 0–151 kBq/ml, volume = 987 mL). In the region of high uptake (typical of tumor hypervascularisation, volume = 303 mL, segmented in green) the averaged activity concentration was 107 kBq/ml (range: 49–421 kBq/mL). (b) Bone SPECT/CT of a patient administered with 937 MBq of ^99m^Tc-labeled diphosphonates. A hot lesion due to a degenerative arthrosis is visible on the left lumbar posterior vertebral articulation (L5-S1) with average activity concentration of 224.5 kBq/mL (range: 172–328 kBq/mL) with an average bone uptake into the SPECT field of view of 60 kBq/mL (SUV_max_ = 46 g/mL).

In vivo validation of ^99m^Tc SPECT/CT quantification was performed on quantitative SPECT/CT of ^99m^Tc-macroaggregated albumin (MAA) liver distribution for dosimetry assessments prior to Y-90 radioembolisation in a series of 10 patients. The recovered total activity in the SPECT field of view (abdominal region centered on the liver) was on average 8% less of the total administered activity. This underestimation can be explained by the fact that part of the administered albumins were not in the field of view of the SPECT/CT acquisition as results of extrahepatic shunts in the pelvic region and the upper part of the lungs. An example is given in Figure 3(a). An example of quantification in a bone ^99m^Tc-labeled diphosphonate SPECT/CT is given in Figure 3(b).

Quantitative methods in ^99m^Tc SPECT can achieve a 5% level of accuracy as reported by Bailey and Willowson, ([3] and references therein). This level of accuracy is compatible with results obtained in most of the phantom configuration tested by us. The tested SPECT/CT device provided a simple and ready-to-use way to achieve absolute quantification of ^99m^Tc at the price of a periodic calibration, once per month as recommended by the vendor, consisting in the measurement of the detector sensitivity with a NIST certified Co-57 source in extrinsic modality (with the low-energy high-resolution collimator on place). The time required by this calibration procedure depends on the source activity; a static acquisition with a total statistic of 5000 k counts is required. Including collimator mounting, energy peaking verification, and point source calibration, no more than an hour is required in our experience when the source activity enables a >5 k counts/s count rate as requested by vendor specifications.

## 4. Conclusion

Absolute quantification in commercial SPECT/CT is now commercially available and a clinical reality. In this work, we tested quantitative accuracy of ^99m^Tc SPECT in different phantom configurations, which progressively mimics anthropomorphic geometry. The tested SPECT/CT scanner provided satisfactory accuracy for both background activity concentration and total activity recovery. Relative lung error evaluated in a NEMA/IEC NU2 phantom study setup was comparable to levels achievable in modern PET scanners using ^18^F-FDG. This last result demonstrates the efficient integration of attenuation and scatter corrections with adequate detector/collimator modeling. Activity recovery in a hot insert is affected by the partial volume effect and needs to be assessed for each clinical setup. Reconstruction parameters influence RC (and thus SUV), and optimization is recommended according to clinical requirements. At fixed reconstruction parameters, RC and hot contrast have shown little variation within the tested counting statistics range. Reliable and ready-to-use SPECT quantification is now available commercially and has the potential to improve monitoring clinical processes and their evolution with therapy, as well as assessing internal radiation dosimetry.

## Figures and Tables

**Table 1:** Background activity concentration (*A*_*c*,bg_), total activity deviation (Δ*A*_tot_), calibration factor (Bg. cal), and coefficient of variation (COV) for 4 SPECT acquisitions of a cylindrical homogeneous phantom.

Homogeneous scan number	*A*_*c*,bg_ (kBq/mL)	Δ*A*_tot_ (%)	Bg. cal	COV%
Scan 1	22	3.9	1.05	13
Scan 2	20	4.1	1.04	12
Scan 3	18	3.5	1.04	12
Scan 4	2	8.6	1.09	38

**Table 2:** SPECT acquisitions of the NEMA/IEC NU2 phantom: Activity concentration in the background (*A*_*c*,bg_) and spherical inserts (*A*_*c*,spheres_), total activity deviation (Δ*A*_tot_), calibration factor (Bg. cal) and coefficient of variation (COV), lung variability (Δ*C* lung), and hot contrast (*Q*_*H*_) for the 22 mm diameter hot insert.

NEMA scan number	*A*_*c*,bg_	*A*_*c*,spheres_	Δ*A*_tot_ (%)	Bg. cal	COV%	Δ*C* lung%	*Q*_*H*_ (%) (22 mm)
Scan 1	23.27	196.87	2.5	1.06	20	5.78	65
Scan 2	18.30	154.82	1.1	1.03	24	5.70	67
Scan 3	12.32	104.20	−1	0.98	28	7.79	65
Scan 4	1.46	12.36	4	1.01	55	20.47	65

**Table 3:** SPECT acquisitions of the liver phantom: activity concentration in the background (*A*_*c*,bg_) and spherical inserts (*A*_*c*,spheres_), total activity deviation (Δ*A*_tot_), calibration factor (Bg. cal) and coefficient of variation (COV), and hot contrast (*Q*_*H*_) for the three inserts.

Liver scan number	*A*_*c*,bg_ kBq/mL	*A*_*c*,Sph._ kBq/mL	Δ*A*_tot_ (%)	Bg. cal	COV%	*Q*_*H*_ 40 mm	*Q*_*H*_ 30 mm	*Q*_*H*_ 20 mm
Scan 1	77.79	404.57	1.9	1.08	8.47	68	51	30
Scan 2	62.13	323.09	0.1	1.07	8.99	67	51	29
Scan 3	42.05	218.70	−0.1	1.06	12.12	67	54	33
Scan 4	4.99	25.95	5.8	1.07	43.25	71	51	33

## References

[1] Rahmim A., Zaidi H. (2008). PET versus SPECT: strengths, limitations and challenges. *Nuclear Medicine Communications*.

[2] Bailey D. L., Willowson K. P. (2014). Quantitative SPECT/CT: SPECT joins PET as a quantitative imaging modality. *European Journal of Nuclear Medicine and Molecular Imaging*.

[3] Bailey D. L., Willowson K. P. (2013). An evidence-based review of quantitative SPECT imaging and potential clinical applications. *Journal of Nuclear Medicine*.

[4] D'Arienzo M., Cicone F., Chiacchiararelli L., Coniglio A., Delaloye A. B., Scopinaro F. (2012). Three-dimensional patient-specific dosimetry in radioimmunotherapy with 90Y-ibritumomab-tiuxetan. *Cancer Biotherapy and Radiopharmaceuticals*.

[5] Patton J. A., Turkington T. G. (2008). SPECT/CT physical principles and attenuation correction. *Journal of Nuclear Medicine Technology*.

[6] Hutton B. F., Buvat I., Beekman F. J. (2011). Review and current status of SPECT scatter correction. *Physics in Medicine and Biology*.

[7] Zeintl J., Vija A. H., Yahil A., Hornegger J., Kuwert T. (2010). Quantitative accuracy of clinical 99mTc SPECT/CT using ordered-subset expectation maximization with 3-dimensional resolution recovery, attenuation, and scatter correction. *Journal of Nuclear Medicine*.

[8] Boellaard R., Krak N. C., Hoekstra O. S., Lammertsma A. A. (2004). Effects of noise, image resolution, and ROI definition on the accuracy of standard uptake values: a simulation study. *Journal of Nuclear Medicine*.

[9] Boellaard R., O'Doherty M. J., Weber W. A. (2010). FDG PET and PET/CT: EANM procedure guidelines for tumour PET imaging: Version 1.0. *European Journal of Nuclear Medicine and Molecular Imaging*.

[10] Bettinardi V., Presotto L., Rapisarda E., Picchio M., Gianolli L., Gilardi M. C. (2011). Physical Performance of the new hybrid PETCT Discovery-690. *Medical Physics*.

